# Long work hours, weekend working and depressive symptoms in men and women: findings from a UK population-based study

**DOI:** 10.1136/jech-2018-211309

**Published:** 2019-02-25

**Authors:** Gillian Weston, Afshin Zilanawala, Elizabeth Webb, Livia A Carvalho, Anne McMunn

**Affiliations:** 1 Research Department of Epidemiology and Public Health, University College London, London, UK; 2 Human Development and Family Science, Oregon State University, Corvallis, Oregon, USA; 3 Department of Research and Policy, Age UK, London, UK; 4 Department of Clinical Pharmacology, William Harvey Research Institute, Queen Mary University of London, London, UK

**Keywords:** depression, gender, mental health, psychosocial factors, work stress

## Abstract

**Background:**

Globalised and 24/7 business operations have fuelled demands for people to work long hours and weekends. Research on the mental health effects of these intensive temporal work patterns is sparse, contradictory or has not considered gender differences. Our objective was to examine the relationship between these work patterns and depressive symptoms in a large nationally representative sample of working men and women in the UK.

**Method:**

The current study analysed data from Understanding Society, the UK Household Longitudinal Study, of 11 215 men and 12 188 women in employment or self-employment at the time of the study. Ordinary least squares regression models, adjusted for potential confounders and psychosocial work factors, were used to estimate depressive symptoms across categories of work hours and weekend work patterns.

**Results:**

Relative to a standard 35–40 hours/week, working 55 hours/week or more related to more depressive symptoms among women (ß=0.75, 95% CI 0.12 to 1.39), but not for men (ß=0.24, 95% CI −0.10 to 0.58). Compared with not working weekends, working most or all weekends related to more depressive symptoms for both men (ß=0.34, 95% CI 0.08 to 0.61) and women (ß=0.50, 95% CI 0.20 to 0.79); however, working some weekends only related to more depressive symptoms for men (ß=0.33, 95% CI 0.11 to 0.55), not women (ß=0.17, 95% CI −0.09 to 0.42).

**Conclusion:**

Increased depressive symptoms were independently linked to working extra-long hours for women, whereas increased depressive symptoms were associated with working weekends for both genders, suggesting these work patterns may contribute to worse mental health.

## Introduction

The demands of operating a 24/7 globalised society is partially met by work patterns that elongate working hours and extend the working week.^1^ In eastern Asian countries the risk of karoshi (death due to overwork) has increased,^2 3^ while across EU countries atypical work hours have become a feature for a significant proportion of people.^4^ In the UK, there are concerns about unregulated and frequently unpaid overtime,^5^ and work-related stress, often linked to workload, accounts for millions of lost working days every year.^6^ Despite this, other than studies on shift work,^7^ few epidemiological studies have considered the impact of temporal work patterns on mental health.

To our knowledge, although weekend working has been associated with poor work-life balance^8^ and work-family conflict,^9 10^ just four studies have examined its relationship to mental health. While three found evidence of job stress or psychological strain among weekend workers relative to weekday workers,^11–13^ in the fourth there was no association between working weekends and depressive symptoms.^14^ Long work hours has attracted more research attention. Recent reviews and a meta-analysis concluded there were some adverse effects of long hours on depressive mood, but these were often small, non-significant, or greater for women.^15–17^

Research has shown that gender plays a significant role in the way that work is organised, experienced and rewarded, not least in terms of occupational segregation, job status, mobility, and inequality in earnings, but also in work attitudes, behaviour and social relations.^18^ There are also suggestions that men and women perceive and respond differently to work demands such as the quantity of work and time-pressures.^19^ Despite this and recommendations that studies about work and health should address gender differences,^20^ most studies on temporal work patterns focus only on men or do not separate men and women in their analysis. Furthermore, although psychosocial work factors link to both working patterns and depression, few of the studies on temporal work patterns take them into account.^21^ Moreover, there is heterogeneity in the way that long hours are defined,^22^ with part-time workers sometimes categorised as the reference group despite part-time work being associated with health problems.^23^ A further limitation is that much of the existing research on UK workers relates to specific workplaces or occupations such as civil servants which, though informative, may not be representative of the general population of workers.^24^

To facilitate generalisability, disaggregate by gender, adjust for a range of covariates including psychosocial work factors, and use the standard working week of 35–40 hours and weekday working as our reference categories, our aim was to investigate the linkages of temporal work patterns with mental health using workers’ data from a large nationally representative sample of the UK population. Our hypothesis was that in comparison to workers who work standard full-time weekly work hours or weekdays, those who do not will have an elevated risk of depression.

## Methods

### Sample

Understanding Society, the UK Household Longitudinal Study (UKHLS) is a longitudinal study following people living in around 40 000 households throughout the UK. It represents the diversity of participants of all ages, ethnicities, and employment status in all four constituent countries. We did not use the first wave of the UKHLS (2009 to 2011) because information on weekend working and work conditions was not available. For the second wave (wave 2, 2010 to 2012) 14 797 men and 14 437 women aged 16 and over were employed or self-employed and not in full-time education; of these 11 215 men and 12 188 women had data on the outcome. UKHLS data are publicly available and data collection was approved by the University of Essex ethics committee.

### Measures

#### Depressive symptoms

Depressive symptoms were measured by the 12-item General Health Questionnaire (GHQ-12), a psychometrically valid tool for studying psychological distress in general and clinical populations.^25^ At wave 2 this tool was administered to participants as part of a computer assisted self-completion questionnaire.

Each item of the GHQ-12 enquires about a specific symptom and whether the participant’s mood state differs from their normal state by asking them to select a response from options: ‘much more than usual’, ‘rather more than usual’, ‘less than usual’, and ‘much less than usual’. The Likert scoring method provided a summed score for the 12 items ranging from 0 (least symptoms) to 36 (most symptoms).^26^

#### Temporal work patterns

We summed the number of hours participants on average worked per week, worked as overtime in a normal week, and worked in any second jobs. Mindful of the lack of consensus in categorising work hours, we chose to adhere to a definition and reference group used in epidemiology studies in the UK: 35–40 hours (standard full-time; reference category), 41–54 hours (long hours), and 55 hours and over (extra-long hours).^23^ To this we added an additional category to account for part-time workers: fewer than 35 hours per week.

Participants were also asked if they ever worked weekends, with three response options: no weekend working (reference category); some weekends; most/all weekends.

#### Covariates

As demographic, socioeconomic and lifestyle factors are determinants of mental health and also associated with work patterns,^27^ we adjusted for: age and age-squared (with the quadratic term added because existing evidence suggests that the trajectory of depressive symptoms in adulthood is u-shaped),^28^ marital status (single, married/cohabiting, separated/divorced/widowed); children in the household (no children, youngest child aged 0–4 years, 5–9 years, 10–15 years); educational attainment (degree or higher, A level or equivalent, GCSE or equivalent, other qualification, no qualifications); equivalised gross monthly household income, created from two variables (gross household income and the modified OECD equivalence scale to adjust for the relative cost of living of households of different compositions).^29^ We adjusted for health behaviours: smoking status (never-smoker, ex-smoker, and current smoker); and a binary indicator of doctor-diagnosed chronic illness (congestive heart failure, coronary heart disease, angina, heart attack or myocardial infarction, stroke, cancer or malignancy, or diabetes).

To account for potential impacts on health from work characteristics such as employment relations, we used the three-category version of the National Statistics Socio-economic Classification (NS-SEC).^30^ This classified people according to their main job: managerial/professional, intermediate, and routine. As the psychosocial work environment is deemed an important link between work and depression^21^ we included the following potential mediators in our models: satisfaction with income (satisfied, neutral satisfaction, dissatisfied); physicality of the job (not at all physical, not very physical, fairly physical, very physical); job satisfaction (satisfied, neutral satisfaction, dissatisfied); and work autonomy (five items measuring autonomy over job task, work pace, work manner, task order, and work hours were summed and reverse coded to give a score of 1 to 20 with higher scores representing greater autonomy).

### Analysis

To account for missing data on exposures and covariates, we applied multiple imputation by chained equations and imputed 33 datasets for each sample of men and women. The imputation model included all analysis variables, and the regressions excluded imputed outcomes (GHQ-12).^31^ Complete case results (shown in the online supplementary tables) were substantially in line with imputed results.

10.1136/jech-2018-211309.supp1 Supplementary file 1

Further to a formal test for gender interactions (not shown, available on request) gender-stratified associations between the atypical work patterns and depressive symptoms were tested using ordinary least squares (OLS) regression models. The first model adjusted for age. The second added more demographic, socioeconomic and health factors. Finally, psychosocial work factors were included. Accounting for the complex design of the UKHLS, study design weights are provided with the data so that the results can be generalised to the UK population. These account for unequal selection probabilities, potential sampling error, non-response of eligible participants, the survey instrument, and the type of observational study. Our analyses used survey commands in Stata V.14. We applied the recommended weight reflecting the cross-sectional study and use of data from the wave 2 self-completion module.

## Results

The distribution of analysis variables in the gender-stratified sample of workers are shown in table 1. In all samples the majority were married, aged 35 years and older, had no children, had no diagnosed chronic illness, and had income satisfaction and job satisfaction.

**Table 1:** Characteristics of men and women by work pattern

Sample	Men (n=11 215)					Women (n=12 188)					Men (n=11 215)				Women (n=12 188)			
Work pattern	Weekly work hours (hours/wk)										Weekend working							
	<35	35–40	41–54	≥55		<35	35–40	41–54	≥55		None	Some	Most/all		None	Some	Most/all	
N	1645	4101	4104	1365		5881	3653	2191	463		3794	4839	2582		6091	3720	2377	
%	14.8	35.9	36.9	12.4		48.7	28.7	18.6	4.0		32.9	44.1	23.0		49.4	30.6	20.0	
Age (years)																		
Mean (95% CI)	45.4	41.3	41.1	42.3		43.6	40.2	39.6	42.3		43.1	42.0	39.8		42.6	42.2	39.3	
	(44.4 to 46.4)	(40.8 to 41.8)	(40.7 to 41.5)	(41.5 to 43.1)		(43.1 to 44.0)	(39.7 to 40.7)	(39.0 to 40.2)	(40.9 to 43.6)		(42.5 to 43.6)	(41.8 to 42.6)	(39.2 to 40.5)		(42.2 to 43.0)	(41.7 to 42.7)	(38.6 to 39.9)	
16–34	31.3	32.9	32.8	28.1		25	35.5	38.6	28.4	***	29.5	30.0	39.6	***	28.2	29.1	39.2	***
≥35	68.7	67.1	67.2	71.9		75	64.5	61.4	71.6		70.5	70.0	60.4		71.8	70.9	60.8	
Marital status					***					***				***				***
Single	27.5	21.9	17.6	15.0		14.5	24.3	26.0	21.7		19.0	18.4	25.9		17.3	19.7	26.0	
Married/cohabit	66.1	73.3	77.8	79.8		73.8	63.5	62.8	63.4		76.5	76.2	69.2		71.4	68.1	61.3	
Seperated/divorced/ widowed	6.4	4.7	4.6	5.1		11.7	12.2	11.2	14.9		4.4	5.4	4.9		11.3	12.2	12.7	
Children in the household					***					***				***				***
None	77.8	65.0	61.9	60.1		52.1	75.1	77.0	77.2		68.0	63.0	65.3		62.0	66.2	67.3	
0–4 years	11.8	17.0	18.7	19.3		18.7	8.4	7.4	7.3		14.9	17.9	18.9		13.3	12.4	14.0	
5–9 years	5.6	8.6	9.1	9.5		14.2	6.1	5.3	4.3		8.4	8.8	7.7		11.3	9.4	6.8	
10–15 years	4.8	9.4	10.3	11.1		15.0	10.4	10.2	11.2		8.6	10.4	8.0		13.4	12.0	11.8	
Education attainment					***					***				***				***
Degree	37.6	40.8	42.6	41.5		37.7	48.3	61.1	65.9		45.2	42.8	31.8		45.3	53.4	37.6	
A level	23.0	23.8	23.9	20.3		19.3	21.2	18.4	11.8		21.4	23.4	25.9		19.7	17.4	21.5	
GCSE	20.6	20.3	20.2	20.9		25.7	19.9	15.2	13.3		18.5	19.5	24.8		21.4	19.1	25.9	
Other qualification	10.0	8.7	9.0	10.9		9.2	6.7	3.5	6.8		8.7	9.3	10.1		7.9	6.0	7.9	
No qualification	8.8	6.3	4.2	6.4		8.2	3.9	1.8	2.3		6.3	4.9	7.3		5.6	4.2	7.2	
NS-SEC occupations					***					***				***				***
Manager/professional	28.7	42.5	47.8	45.5		27.8	47.0	61.8	65.2		50.4	44.8	27.8		41.3	49.0	28.7	
Intermediate	29.6	21.7	18.8	22.9		26.2	27.8	16.7	19.7		18.5	22.7	25.2		29.1	20.6	20.0	
Routine	41.8	35.8	33.4	31.7		46.1	25.2	21.4	15.1		31.2	32.6	47.0		29.6	31.3	51.3	
Equivalised household income					***					***				***				***
Quintile 1 (lowest)	16.7	5.7	4.1	6.8		12.1	4.6	3.0	4.3		5.4	5.8	10.9		7.0	7.0	11.8	
Quintile 2	20.7	13.7	10.7	9.2		20.7	10.7	7.9	7.4		12.3	11.8	16.3		14.4	13.0	19.2	
Quintile 3	20.7	22.4	18.3	16.2		23.4	21.0	15.1	11.0		20.8	17.9	22.3		21.2	19.0	21.8	
Quintile 4	19.7	29.3	27.8	24.8		23.5	30.9	27.0	20.7		26.5	27.4	25.8		27.9	25.1	23.7	
Quintile 5 (highest)	22.2	29.0	39.1	43.1		20.3	32.7	47.0	56.6		34.9	37.0	24.7		29.6	35.9	23.4	
Chronic illness					***					***				***				**
None diagnosed	74.3	81.4	82.6	81.5		80.8	84.6	85.7	83.4		79.9	80.1	83.4		81.7	83.7	84.8	
Diagnosed	25.7	18.6	17.4	18.5		19.2	15.4	14.3	16.6		20.1	19.9	16.6		18.3	16.3	15.2	
Smoker status					***									***				***
Non-smoker	35.7	41.0	39.9	35.0		45.6	47.0	45.3	45.0		42.3	37.5	37.5		48.9	44.2	41.2	
Ex-smoker	41.8	36.4	37.6	36.8		34.3	31.9	27.0	37.5		37.4	39.1	35.4		33.3	35.6	30.4	
Smoker	22.5	22.6	22.5	28.3		20.2	21.0	22.0	17.5		20.4	23.4	27.1		17.8	20.2	28.4	
Income satisfaction					***					***				***				***
Satisfied	55.4	57.9	62.5	62.6		56.2	59.2	64.8	64.9		61.3	61.1	54.9		60.7	60.1	53.2	
Neutral	13.7	14.0	12.5	12.1		12.7	11.4	10.2	8.4		12.3	13.4	13.9		10.9	11.6	14.0	
Dissatisfied	30.8	28.2	25.0	25.4		31.1	29.3	25.0	26.7		26.3	25.4	31.2		28.4	28.3	32.9	
Job physicality					***					***				***				***
Not at all	26.2	23.3	24.7	32.3		22.1	16.5	19.1	28.1		18.7	25.1	35.2		14.4	21.0	33.0	
Not very	41.7	35.8	37.2	35.4		43.3	35.4	36.7	42.6		33.7	37.6	41.2		36.4	40.3	47.3	
Fairly	19.7	24.6	25.4	23.4		21.4	29.8	30.4	18.5		28.3	24.7	16.6		30.0	25.4	14.0	
Very physical	12.4	16.3	12.7	9.0		13.2	18.3	13.8	10.8		19.3	12.6	6.9		19.01	13.3	5.7	
Job satisfaction										*				**				**
Satisfied	77.7	75.1	77.6	80.0		79.7	80.5	79.9	78.1		74.4	79.1	76.7		80.9	80.0	77.0	
Neutral	8.5	8.5	7.9	6.6		7.0	6.2	4.5	6.1		9.3	7.1	8.2		5.8	5.7	8.0	
Dissatisfied	13.8	16.5	14.5	13.4		13.4	13.3	15.6	15.8		16.3	13.9	15.2		13.2	14.4	14.9	
Work autonomy					**					**				***				**
Mean (95% CI)	10.9	11.4	11.9	12.5		10.3	11.1	11.5	11.9		11.6	12.0	11.2		10.8	11.0	10.4	
	(10.6 to 11.2)	(11.3 to 11.6)	(11.8 to 12.1)	(12.3 to 12.8)		(10.2 to 10.4)	(10.9 to 11.2)	(11.3 to 11.6)	(11.4 to 12.3)		(11.4 to 11.7)	(11.8 to 12.1)	11.0 to 11.5)		(10.7 to 10.9)	(10.8 to 11.2)	(10.2 to 10.6)	

Figures are percentages unless stated otherwise. Percentages are weighted. Sample sizes are unweighted.

*P<0.05; **P<0.01; ***P<0.001.

NS-SEC, National Statistics Socio-Economic Classification.

### Weekly work hours

Men tended to work longer hours than women: almost half the men worked longer than the standard 35–40 hours/week compared with less than a quarter of the women, and nearly half of women worked part-time (<35 hours/week) compared with 15% of men. Education, income and occupational classification were positively associated with work hours for men and women; however, whereas having children and being married were negatively associated with long work hours for women, the opposite was found for men. Generally, part-time workers were the most likely to be in routine jobs and have the least work autonomy, whereas those working extra-long hours (≥55 hours/week) had the highest household incomes and greatest work autonomy.

### Weekend working

More men than women worked weekends: over two-thirds of men and half of the women worked at weekends. Of these, the majority worked some rather than most/all weekends. In contrast to our findings above, although married men worked the longest hours they were not more likely to work weekends; and generally, among both men and women, age, education, income and occupational classification were negatively associated with weekend work. There were also differences relating to the frequency of weekend working, with those working most/all weekends more likely to be in routine jobs, in the least physically active jobs, with the most income dissatisfaction, the least job satisfaction, and the lowest work autonomy. Both men and women working some weekends had more work autonomy than those in either of the other categories.

### Depressive symptoms

Table 2 presents unadjusted mean depressive symptoms for the temporal work patterns, covariates, and work conditions. Compared with the reference categories (35–40 hours/week; no weekends), there was no difference in the number of depressive symptoms for men working fewer or longer hours or any weekends, whereas women working ≥55 hours/week and those working most/all weekends had significantly more symptoms.

**Table 2:** Unadjusted mean depressive symptoms for work arrangements and covariates for men and women

Temporal work patterns	Men	Women
	Mean GHQ-12 (95% CI)	Mean GHQ-12 (95% CI)
Weekly work hours (hours/week)		
<35	10.1 (9.7 to 10.5)	11.1 (10.9 to 11.2)
35–40†	10.1 (9.9 to 10.3)	11.0 (10.8 to 11.2)
41–54	10.0 (9.8 to 10.2)	11.2 (10.9 to 11.5)
≥55	10.1 (9.8 to 10.4)	11.8 (11.1 to 12.4)*
Weekend work		
No weekends†	9.9 (9.8 to 10.1)	10.9 (10.7 to 11.1)
Some weekends	10.1 (10.0 to 10.3)	11.1 (10.9 to 11.3)
Most/all weekends	10.1 (9.9 to 10.4)	11.5 (11.2 to 11.8)***
Covariates		
Age (years)		
16–34	9.8 (9.6 to 9.9)***	10.8 (10.6 to 11.1)**
≥35†	10.2 (10.1 to 10.3)	11.2 (11.1 to 11.3)
Marital status		
Single	9.8 (9.5 to 10.0)*	11.0 (10.7 to 11.3)
Married†	10.1 (10.0 to 10.2)	11.0 (10.8 to 11.1)
Separated/divorced/widowed	10.5 (10.0 to 10.9)	11.9 (11.5 to 12.2)***
Children in the household		
None†	10.0 (9.8 to 10.1)	11.0 (10.8 to 11.1)
Aged 0–4 years	10.0 (9.8 to 10.2)	11.2 (10.9 to 11.5)
Aged 5–9 years	10.4 (10.1 to 10.8)*	11.0 (10.7 to 11.4)
Aged 10–15 years	10.6 (10.3 to 11.0)***	11.5 (11.2 to 11.8)**
Educational attainment		
Degree (or higher)†	10.3 (10.1 to 10.4)	11.0 (10.8 to 11.1)
A levels (or equivalent)	10.0 (9.8 to 10.3)	11.0 (10.8 to 11.3)
GCSEs (or equivalent)	9.9 (9.7 to 10.1)**	11.3 (11.1 to 11.6)*
Other qualification	10.0 (9.7 to 10.3)	11.5 (11.1 to 11.9)*
No qualifications	9.7 (9.3 to 10.2)*	10.8 (10.3 to 11.3)
NS-SEC occupations		
Managerial/professional†	10.2 (10.0 to 10.3)	11.1 (10.9 to 11.3)
Intermediate	10.2 (10.0 to 10.5)	10.9 (10.7 to 11.2)
Routine	9.9 (9.7 to 10.0)**	11.2 (10.9 to 11.4)
Equivalised household income		
1st quintile	10.6 (10.2 to 11.0)**	11.7 (11.4 to 12.2)***
2nd quintile	10.3 (10.0 to 10.5)*	11.5 (11.2 to 11.8)***
3rd quintile	10.1 (9.8 to 10.3)	10.9 (10.7 to 11.2)
4th quintile	10.1 (9.9 to 10.3)	11.0 (10.8 to 11.3)
5th quintile (highest amount)†	9.9 (9.7 to 10.1)	10.9 (10.7 to 11.1)
Chronic illness:		
Not diagnosed†	10.0 (9.8 to 10.1)	11.0 (10.8 to 11.1)
Diagnosed	10.5 (10.3 to 10.8)***	11.7 (11.4 to 12.0)***
Smoker status:		
Non-smoker†	9.9 (9.7 to 10.0)	10.7 (10.5 to 10.9)
Ex-smoker	10.0 (9.9 to 10.2)	11.1 (10.9 to 11.3)***
Smoker	10.5 (10.2 to 10.7)***	11.9 (11.6 to 12.2)***
Psychosocial work conditions		
Satisfaction with income		
Satisfied†	9.1 (9.0 to 9.3)	10.0 (9.8 to 10.1)
Neutral satisfaction	10.2 (9.9 to 10.5)***	11.5 (11.2 to 11.9)***
Dissatisfied	12.0 (11.8 to 12.3)***	13.1 (12.9 to 13.4)***
Job physicality:		
Not at all physical†	9.7 (9.5 to 9.9)	11.0 (10.7 to 11.3)
Not very physical	10.0 (9.8 to 10.2)*	11.0 (10.8 to 11.2)
Fairly physical	10.4 (10.1 to 10.6)***	11.1 (10.8 to 11.3)
Very physical	10.5 (10.2 to 10.8)***	11.4 (11.1 to 11.7)*
Job satisfaction		
Satisfied†	9.5 (9.3 to 9.6)	10.5 (10.3 to 10.6)
Neutral satisfaction	11.2 (10.8 to 11.5)***	12.7 (12.2 to 13.2)***
Dissatisfied	12.6 (12.3 to 13.0)***	14.0 (13.6 to 14.3)***

† Denotes reference category. Means are weighted.

*P<0.05; **P<0.01; ***P<0.001.

GHQ-12, 12-item General Health Questionnaire; NS-SEC, National Statistics Socio-Economic Classification.

Generally, in both genders, relative to the reference categories, the number of symptoms were higher for older workers, smokers, and participants with the lowest household incomes, chronic illness, job and income dissatisfaction, very physical jobs and the lowest work autonomy. In terms of gender differences, men who were single, in routine occupations, and had GCSE or no qualifications had fewer depressive symptoms; whereas women who were separated/divorced/widowed, had older children, and had GCSE and ‘other qualifications’ had the highest number of symptoms.

### Temporal work patterns and depressive symptoms

#### Weekly work hours

As table 3 shows, in all models, women working extra-long hours (≥55 hours/week) had more depressive symptoms relative to women working standard hours (35–40 hours/week). Men working part-time (<35 hours/week) had significantly more symptoms than men working standard hours, but this association was attenuated by the inclusion of education, income and chronic illness. In all models, relative to working standard hours, there was no difference in symptoms for women working part-time (<35 hours/week) or long hours (41–54 hours/week), or among men working long hours (41–54 hours/week) or extra-long hours (≥55 hours/week).

**Table 3:** Associations between weekly work hours and depressive symptoms

	Men						Women					
	(1) Age		(2)+SEP+health†		(3)+mediators‡		(1) Age		(2)+SEP+health†		(3)+mediators‡	
	Coef	95% CI	Coef	95% CI	Coef	95% CI	Coef	95% CI	Coef	95% CI	Coef.	95% CI
Work schedule (reference: 35–40 hours/week												
<35 hours/week	0.41*	0.05 to 0.77	0.29	−0.06 to 0.65	0.25	−0.07 to 0.58	0.04	−0.22 to 0.29	−0.06	−0.33 to 0.21	−0.07	−0.33 to 0.19
41–54 hours/week	−0.15	−0.39 to 0.09	−0.10	−0.35 to 0.14	0.04	−0.19 to 0.27	0.18	−0.17 to 0.53	0.20	−0.14 to 0.55	0.26	−0.05 to 0.57
≥55 hours/week	−0.03	−0.38 to 0.32	−0.002	−0.36 to 0.35	0.24	−0.10 to 0.58	0.73*	0.04 to 1.41	0.76*	0.08 to 1.43	0.75*	0.12 to 1.39
Age (continuous)												
Age	0.23***	0.18 to 0.27	0.21***	0.16 to 0.27	0.15***	0.10 to 0.20	0.17***	0.12 to 0.22	0.18***	0.11 to 0.24	0.12***	0.05 to 0.18
Age2	−0.003***	−0.003 to -0.002	−0.003***	−0.003 to -0.002	−0.002***	−0.002 to -0.001	−0.002***	−0.003 to -0.001	−0.002***	−0.003 to -0.001	−0.001***	−0.002 to -0.001
Marital status (reference: married)												
Single/never married			−0.05	−0.40 to 0.31	−0.03	−0.37 to 0.31			0.20	−0.18 to 0.59	0.02	−0.35 to 0.39
Separated/divorced/widowed			0.24	−0.20 to 0.69	0.14	−0.26 to 0.55			0.66***	0.30 to 1.02	0.21	−0.13 to 0.54
Children in household (reference: none)												
0–4 years			−0.30	−0.63 to 0.03	−0.17	−0.49 to 0.14			0.25	−0.13 to 0.63	0.29	−0.07 to 0.66
5–9 years			−0.02	−0.44 to 0.40	0.03	−0.36 to 0.42			−0.14	−0.55 to 0.27	−0.017	−0.40 to 0.37
10–15 years			0.20	−0.17 to 0.57	0.28	−0.07 to 0.62			0.12	−0.24 to 0.48	0.24	−0.09 to 0.58
Education attainment (reference: degree)												
A level			−0.18	−0.48 to 0.12	−0.21	−0.50 to 0.08			0.07	−0.25 to 0.38	0.003	−0.30 to 0.30
GCSE			−0.51***	−0.81 to -0.21	−0.46**	−0.74 to -0.18			0.21	−0.12 to 0.53	0.20	−0.11 to 0.50
Other qualification			−0.50**	−0.87 to -0.12	−0.46*	−0.82 to -0.11			0.38	−0.11 to 0.86	0.35	−0.10 to 0.80
No qualifications			−0.75**	−1.27 to -0.24	−0.57*	−1.06 to -0.08			−0.24	−0.81 to 0.32	−0.14	−0.67 to 0.40
NS-SEC (reference: manager/professional)												
Intermediate			0.03	−0.27 to 0.33	0.06	−0.22 to 0.35			−0.18	−0.48 to 0.12	−0.30*	−0.59 to 0.01
Routine			−0.27	−0.55 to 0.02	−0.51***	−0.81 to -0.21			−0.12	−0.46 to 0.22	−0.31	−0.65 to 0.03
Equivalised household income (reference: 5th quintile)												
4th quintile			0.34*	0.05 to 0.62	−0.06	−0.34 to 0.21			0.18	−0.13 to 0.48	−0.12	−0.40 to 0.16
3rd quintile			0.44**	0.12 to 0.76	−0.13	−0.43 to 0.17			0.002	−0.33 to 0.33	−0.50**	−0.81 to -0.19
2nd quintile			0.66***	0.30 to 1.02	−0.13	−0.49 to 0.22			0.55**	0.15 to 0.95	−0.17	−0.55 to 0.20
1st quintile (lowest)			0.93***	0.43 to 1.42	0.25	−0.21 to 0.71			0.70**	0.17 to 1.24	−0.01	−0.50 to 0.48
Smoker status (reference: non-smoker)												
Ex-smoker			0.20	−0.03 to 0.43	0.15	−0.06 to 0.37			0.36**	0.11 to 0.60	0.18	−0.05 to 0.41
Smoker			0.71***	0.43 to 1.00	0.44**	0.17 to 0.70			1.06***	0.73 to 1.39	0.66***	0.35 to 0.97
Chronic illness (reference: none diagnosed)												
Diagnosed condition			0.67***	0.38 to 0.97	0.55***	0.28 to 0.83			0.76***	0.43 to 1.09	0.54***	0.24 to 0.85
Income satisfaction (reference: satisfied)												
Neutral					0.87***	0.55 to 1.20					1.43***	1.09 to 1.78
Dissatisfied					2.42***	2.15 to 2.68					2.75***	2.47 to 3.02
Job physicality (reference: not at all physical)												
Not very physical					0.22	−0.03 to 0.47					0.23	−0.07 to 0.53
Fairly physical					0.53***	0.23 to 0.83					0.23	−0.10 to 0.56
Very physical					0.41*	0.03 to 0.79					0.48*	0.08 to 0.87
Job satisfaction (reference: satisfied)												
Neutral					1.16***	0.80 to 1.53					1.81***	1.33 to 2.29
Dissatisfied					2.40***	2.04 to 2.76					2.91***	2.52 to 3.30
Work autonomy (continuous)												
Work autonomy					−0.08**	−0.10 to -0.05					−0.06***	−0.08 to -0.03
Constant	5.51***	4.47 to 6.56	5.53***	4.34 to 6.72	6.53***	5.35 to 7.72	7.62***	6.47 to 8.77	6.82***	5.36 to 8.28	7.45***	6.02 to 8.88

Survey weights are applied.

*P<0.05; **P<0.01; ***P<0.001.

†Model is adjusted for age, age2, marital status, children in household, education attainment, NS-SEC, equivalised household income, smoker status and chronic illness.

‡Model is also adjusted for income satisfaction, job physicality, job satisfaction, and work autonomy.

Coef, ß coefficient; NS-SEC, National Statistics Socio-Economic Classification.

#### Weekend working

As shown in table 4, compared with no weekend working, women working most/all weekends had significantly more depressive symptoms in the minimally adjusted model, and this was only slightly attenuated on further adjustment, whereas there was no association with some weekends, even after adjustment. Among men, only after accounting for work conditions was weekend working (most/all and some weekends) associated with significantly more depressive symptoms.

**Table 4:** Associations between weekend working and depressive symptoms

	Men						Women					
	(1) Age		(2)+SEP+health†		(3)+mediators‡		(1) Age		(2)+SEP+health†		(3)+mediators‡	
	Coef	95% CI	Coef	95% CI	Coef	95% CI	Coef	95% CI	Coef	95% CI	Coef	95% CI
Work schedule (reference: no weekends)												
Some weekends	0.15	−0.09 to 0.38	0.14	−0.10 to 0.37	0.33**	0.11 to 0.55	0.20	−0.07 to 0.46	0.17	−0.10 to 0.43	0.17	−0.09 to 0.42
Most/all weekends	0.24	−0.04 to 0.52	0.23	−0.06 to 0.51	0.34*	0.08 to 0.61	0.70***	0.38 to 1.01	0.55***	0.24 to 0.87	0.50**	0.20 to 0.79
Age (continuous)												
Age	0.20***	0.16 to 0.25	0.20***	0.15 to 0.25	0.14***	0.09 to 0.19	0.19***	0.13 to 0.24	0.19***	0.12 to 0.25	0.13***	0.06 to 0.19
Age2	−0.002***	−0.003 to -0.002	−0.002***	−0.003 to -0.002	−0.002***	−0.002 to -0.001	−0.002***	−0.003 to -0.002	−0.002***	−0.003 to -0.002	−0.001***	−0.002 to -0.001
Marital status (reference: married)												
Single/never married			−0.04	−0.39 to 0.32	−0.04	−0.38 to 0.30			0.22	−0.17 to 0.61	0.04	−0.33 to 0.41
Separated/divorced/ widowed			0.23	−0.21 to 0.68	0.13	−0.28 to 0.53			0.67***	0.31 to 1.03	0.23	−0.10 to 0.56
Children in household (reference: none)												
0–4 years			−0.31	−0.64 to 0.03	−0.19	−0.50 to 0.13			0.19	−0.17 to 0.55	0.22	−0.13 to 0.56
5–9 years			−0.02	−0.44 to 0.40	0.03	−0.36 to 0.42			−0.17	−0.57 to 0.23	−0.07	−0.44 to 0.30
10–15 years			0.18	−0.19 to 0.56	0.26	−0.09 to 0.61			0.10	−0.26 to 0.45	0.21	−0.12 to 0.54
Education attainment (reference: degree)												
A level			−0.21	−0.51 to 0.09	−0.22	−0.51 to 0.07			0.06	−0.26 to 0.37	−0.01	−0.31 to 0.29
GCSE			−0.53***	−0.83 to -0.23	−0.46**	−0.74 to -0.19			0.18	−0.14 to 0.51	0.17	−0.13 to 0.48
Other qualification			−0.53**	−0.90 to -0.15	−0.47*	−0.83 to -0.11			0.37	−0.12 to 0.85	0.34	−0.11 to 0.79
No qualifications			−0.78**	−1.30 to -0.26	−0.56*	−1.05 to -0.08			−0.26	−0.83 to 0.30	−0.16	−0.69 to 0.38
NS-SEC (reference: manager/professional)												
Intermediate			0.04	−0.26 to 0.33	0.05	−0.23 to 0.34			−0.22	−0.52 to 0.08	−0.34*	−0.63 to 0.05
Routine			−0.28	−0.57 to 0.01	−0.53***	−0.83 to -0.23			−0.25	−0.59 to 0.09	−0.42*	−0.76 to -0.08
Equivalised household income (reference: 5th quintile)												
4th quintile			0.35*	0.07 to 0.63	−0.06	−0.33 to 0.21			0.14	−0.16 to 0.44	−0.16	−0.44 to 0.12
3rd quintile			0.47**	0.15 to 0.79	−0.11	−0.41 to 0.19			−0.05	−0.37 to 0.28	−0.55***	−0.86 to -0.25
2nd quintile			0.72***	0.35 to 1.08	−0.11	−0.46 to 0.25			0.48*	0.09 to 0.88	−0.24	−0.61 to 0.12
1st quintile (lowest)			1.00***	0.51 to 1.50	0.29	−0.17 to 0.75			0.60*	0.08 to 1.13	−0.11	−0.59 to 0.37
Smoker status (reference: non-smoker)												
Ex-smoker			0.20	−0.03 to 0.43	0.15	−0.06 to 0.36			0.35**	0.11 to 0.59	0.18	−0.05 to 0.41
Smoker			0.71***	0.42 to 0.99	0.43**	0.16 to 0.70			1.04***	0.71 to 1.37	0.65***	0.34 to 0.96
Chronic illness (reference: none diagnosed)												
Diagnosed condition			0.66***	0.36 to 0.96	0.53***	0.25 to 0.81			0.77***	0.44 to 1.11	0.56***	0.25 to 0.86
Income satisfaction (reference: satisfied)												
Neutral					0.87***	0.54 to 1.19					1.42***	1.08 to 1.76
Dissatisfied					2.41***	2.15 to 2.68					2.74***	2.47 to 3.01
Job physicality (reference: not at all physical)												
Not very physical					0.23	−0.02 to 0.49					0.25	−0.05 to 0.55
Fairly physical					0.56***	0.26 to 0.86					0.28	−0.06 to 0.61
Very physical					0.46*	0.08 to 0.85					0.53**	0.14 to 0.93
Job satisfaction (reference: satisfied)												
Neutral					1.18***	0.81 to 1.55					1.77***	1.29 to 2.24
Dissatisfied					2.41***	2.05 to 2.77					2.91***	2.52 to 3.30
Work autonomy (continuous)												
Work autonomy					−0.08***	−0.10 to -0.05					−0.05***	−0.08 to -0.03
Constant	5.77***	4.77 to 6.77	5.64***	4.49 to 6.80	6.55***	5.40 to 7.70	7.17***	6.02 to 8.32	6.59***	5.15 to 8.04	7.21***	5.81 to 8.62

Survey weights are applied.

*P<0.05; **P<0.01; ***P<0.001.

†Model is adjusted for age, age2, marital status, children in household, education attainment, NS-SEC, equivalised household income, smoker status and chronic illness.

†Model is also adjusted for income satisfaction, job physicality, job satisfaction, and work autonomy.

Coef, ß coefficient; NS-SEC, National Statistics Socio-Economic Classification.

## Discussion

### Main findings

Atypical temporal work patterns were associated with small but statistically significant elevations in depressive symptoms in a nationally representative sample of working people in the UK, which was unrestricted by occupations, employer, age or sex, and which took account of psychosocial work factors.

Our results suggest that depressive symptoms were slightly higher among weekend workers compared with non-weekend workers. Furthermore, among women, there was a suggestion of a dose–response-type pattern, while among men, psychosocial work conditions appeared to play a role in the linkage between weekend working and depressive symptoms. Men who worked weekends had higher job satisfaction than those who did not work weekends, so higher levels of depressive symptoms emerged once this was taken into account. This study extends the limited amount of published research on weekend working, which though not gender-stratified, had shown higher emotional exhaustion and job stress among weekend workers,^11,12^ and in an occupation specific sample, had found higher psychological strain linked to the frequency of weekend working.^13^ In contrast, a national cross-sectional study of employees in France, which had disaggregated by gender, found no association between weekend working and depressive symptoms.^14^ However, it restricted its definition of weekend workers to those working ‘at least one Sunday or Saturday every week’, resulting in a sample of 17% women and 19% men with this work pattern. Our analyses differentiated between ‘most/all’ and ‘some’ weekend working, resulting in a less heterogeneous reference group of non-weekend workers, and a greater proportion overall of weekend workers (67% of men and 51% women) in our sample.

Our results also suggest that among women, but not men, working extra-long hours (≥55 hours/week) is linked to more depressive symptoms than working standard full-time hours, which corresponds with previous findings of stronger associations between long work hours and depressive disorders for women than men.^17 23^ We also found elevated symptoms of depression among men working the fewest hours (<35 hours/week), but this effect was explained by socioeconomic and physical health disadvantages among this group. Due to the cross-sectional nature of our study we cannot confirm that men were selected into part-time work because of their health; however, it is noteworthy that previous research found that individuals with health problems were more likely to work part-time rather than full-time.^32^

### Mechanisms and implications

Potential pressures arising from working against social and labour-force norms might explain why there were elevated depressive symptoms among those women working extra-long hours and most/all weekends. Consistent with this suggestion are reports that it is usual in UK society for men to work longer hours^33^ and weekends^34^; indeed in our sample, only 4% of women worked extra-long hours compared with three times as many men, and about 33% more men than women worked at weekends. Another explanation for the differences we found for men and women might relate to the gendered nature of some work: women have been found to work longer hours in male-dominated occupations^35^; and women working weekends tend to be concentrated in low-paid service sector jobs.^36^ Such jobs, when combined with frequent or complex interactions with the public or clients, have been linked to higher levels of depression.^37^ Our finding of more depressive symptoms among women working extra-long hours might also be explained by the potential double-burden experienced by women when their long hours in paid work are added on to their time in domestic labour. Previous studies have found that once unpaid housework and caring is accounted for, women work longer than men on average,^38^ and that this has been linked to poorer physical health.^39^ An investigation into the combined effects of domestic labour and work patterns was beyond the scope of this paper, but this could be an interesting avenue for future research.

### Strengths and limitations

Although previous studies are informative about temporal work patterns for specific groups of workers or their physical health, our study is unique in focusing on a large, nationally representative, heterogeneous sample of workers of all ages (16+) with results that are generalisable to the UK. This sample enabled us to analyse data on women as well as men, reflecting the participation of both genders in the workforce and their different experiences of paid work.

Depressive symptoms were measured using the GHQ-12 scale, a validated standard measure of common mental health, but due to the cross-sectional design of our study, we cannot rule out the possibility of pre-existing symptoms. However, we consider it unlikely that depressed workers would select into long hours and weekend schedules. Indeed, longitudinal studies found that workers who experienced deterioration in their mental health adapted to it by reducing their work hours and changing their work patterns and jobs^40^; also, long work hours were a causal factor for depressive symptoms among civil servants.^23 41^

## Conclusion

Our study shows a link between atypical temporal work patterns and depressive symptoms, but there are gender differences in these associations. The poorest mental health is experienced by women working extra-long hours and most/all weekends, and by men with poor psychosocial work conditions working at weekends. Our findings should encourage employers and policymakers to consider interventions aimed at reducing women’s burdens without restricting their full participation in the workforce, and at improving psychosocial work conditions.

**What is already known on this topic**

- The global and gig economies are driving the need for people to work atypical temporal schedules, like weekends and long hours. Some of these schedules have been associated with physical health disorders but less is known about their relationships with mental health.
- Existing studies focus mainly on men and specific occupations, but rarely examine the effects of weekend working or account for psychosocial working conditions.

**What this study adds**

- Results from this population-based study show gender differences in the associations between atypical temporal work schedules and depressive symptoms, with women who work extra-long hours and most/all weekends experiencing the poorest mental health.
- Men working weekends also experience poorer mental health when their psychosocial conditions are poor.
