# Supplementary material for: Long work hours, weekend working and depressive symptoms in men and women: findings from a UK population-based study
Source: J Epidemiol Community Health. 2019 Feb 25;73(5):465–74. doi: 10.1136/jech-2018-211309 (PMC6581113; doi:10.1136/jech-2018-211309)
Supplement: Supplementary file 1 [file jech-2018-211309supp001.docx]

**Online supplementary tables providing the complete case results.**

| **Table S1: Characteristics of men and women by work pattern (results for complete case analysis)** | | | | | | | | | | | | | | | | | | |
| --- | --- | --- | --- | --- | --- | --- | --- | --- | --- | --- | --- | --- | --- | --- | --- | --- | --- | --- |
| **Sample** | **Men (N=9945)** | | | | | **Women (N=11209)** | | | | | **Men (N=9945)** | | | | **Women (N=11209)** | | | |
| **Work pattern** | **Weekly work hours (hrs/wk)** | | | | | | | | | | **Weekend working** | | | | | | | |
|  | **<35** | **35-40** | **41-54** | **≥55** |  | **<35** | **35-40** | **41-54** | **≥55** |  | **None** | **Some** | **Most/all** |  | **None** | **Some** | **Most/all** |  |
| N | 1429 | 3653 | 3661 | 1202 |  | 5485 | 3320 | 1984 | 420 |  | 3379 | 4291 | 2275 |  | 5677 | 3395 | 2137 |  |
| % | 14.8 | 35.9 | 37.0 | 12.3 |  | 49.4 | 28.2 | 18.4 | 4.0 |  | 33.2 | 44.0 | 22.9 |  | 49.9 | 30.4 | 19.7 |  |
| **Age (years):** |  |  |  |  | *** |  |  |  |  | *** |  |  |  | *** |  |  |  | *** |
| Mean (95%CI) | 46.1 | 41.7 | 41.5 | 42.6 |  | 43.8 | 40.8 | 40.0 | 42.4 |  | 43.5 | 42.5 | 40.4 |  | 42.9 | 42.5 | 39.8 |  |
|  | 45.0 | 41.2 | 41.0 | 41.8 |  | 43.3 | 40.3 | 39.4 | 41.0 |  | 42.9 | 42.1 | 39.7 |  | 42.5 | 42.0 | 39.1 |  |
| 16-34 | 47.2 | 42.2 | 41.9 | 43.4 |  | 44.2 | 41.3 | 40.7 | 43.8 |  | 44.1 | 43.0 | 41.1 |  | 43.3 | 43.0 | 40.5 |  |
| ≥35 | 29.6 | 31.3 | 31.5 | 27.5 |  | 24.3 | 33.3 | 36.9 | 28.2 | *** | 28.2 | 28.8 | 38.0 | *** | 27.0 | 27.9 | 37.5 | *** |
| **Marital status:** |  |  |  |  | *** |  |  |  |  |  |  |  |  | *** |  |  |  |  |
| Single | 26.3 | 21.0 | 16.3 | 14.2 |  | 13.4 | 22.7 | 25.0 | 21.5 |  | 77.4 | 76.6 | 70.7 |  | 72.1 | 68.8 | 62.3 |  |
| Married/cohabit | 66.9 | 74.1 | 78.7 | 80.2 |  | 74.7 | 64.4 | 63.0 | 63.0 |  | 18.1 | 17.5 | 24.1 |  | 16.1 | 18.6 | 24.2 |  |
| Sep/div/widow | 6.8 | 4.9 | 5.0 | 5.6 |  | 11.9 | 13.0 | 12.0 | 15.6 |  | 4.5 | 5.9 | 5.2 |  | 11.8 | 12.6 | 13.5 |  |
| **Children in the household:** |  |  |  |  | *** |  |  |  |  | *** |  |  |  | *** |  |  |  | *** |
| None | 77.7 | 63.9 | 61.4 | 60.7 |  | 51.0 | 74.2 | 76.5 | 76.9 |  | 67.6 | 62.3 | 64.6 |  | 61.0 | 65.4 | 65.8 |  |
| 0-4 years | 11.8 | 17.6 | 18.9 | 19.2 |  | 19.2 | 8.6 | 7.8 | 7.7 |  | 15.0 | 18.3 | 19.2 |  | 13.6 | 12.9 | 14.7 |  |
| 5-9 years | 5.3 | 8.7 | 9.2 | 8.8 |  | 14.6 | 6.4 | 5.3 | 4.3 |  | 8.4 | 8.6 | 8.0 |  | 11.7 | 9.6 | 7.1 |  |
| 10-15 years | 5.2 | 9.8 | 10.5 | 11.3 |  | 15.3 | 10.8 | 10.4 | 11.1 |  | 9.0 | 10.7 | 8.2 |  | 13.7 | 12.1 | 12.4 |  |
| **Education attainment:** |  |  |  |  |  |  |  |  |  |  |  |  |  | *** |  |  |  | *** |
| Degree | 38.1 | 40.5 | 43.3 | 42.1 | *** | 37.6 | 48.4 | 60.3 | 66.6 | *** | 45.0 | 43.5 | 32.0 |  | 45.2 | 53.1 | 37.2 |  |
| A level | 22.9 | 23.8 | 23.5 | 20.7 |  | 19.2 | 21.1 | 18.5 | 11.2 |  | 21.4 | 23.3 | 25.7 |  | 19.7 | 17.5 | 21.0 |  |
| GCSE | 20.7 | 20.3 | 20.1 | 19.7 |  | 26.0 | 19.9 | 15.6 | 13.3 |  | 18.6 | 19.2 | 24.4 |  | 21.6 | 19.3 | 26.5 |  |
| Other qualification | 10.1 | 8.9 | 9.0 | 11.1 |  | 9.2 | 6.7 | 3.6 | 6.6 |  | 8.8 | 9.3 | 10.4 |  | 7.9 | 6.2 | 7.9 |  |
| No qualification | 8.2 | 6.4 | 4.1 | 6.4 |  | 8.0 | 3.9 | 2.0 | 2.3 |  | 6.2 | 4.7 | 7.5 |  | 5.7 | 4.0 | 7.5 |  |
| **NS-SEC occupations:** |  |  |  |  | *** |  |  |  |  | *** |  |  |  | *** |  |  |  | *** |
| Manager/professional | 29.1 | 43.2 | 48.7 | 45.8 |  | 28.2 | 47.3 | 62.4 | 65.5 |  | 51.0 | 46.0 | 27.7 |  | 41.2 | 49.4 | 29.3 |  |
| Intermediate | 30.6 | 21.4 | 18.6 | 22.4 |  | 26.3 | 27.9 | 16.3 | 19.6 |  | 18.5 | 22.5 | 25.4 |  | 29.1 | 20.5 | 19.8 |  |
| Routine | 40.3 | 35.4 | 32.8 | 31.8 |  | 45.5 | 24.8 | 21.3 | 14.9 |  | 30.5 | 31.5 | 47.0 |  | 29.7 | 30.1 | 50.9 |  |
| **Equivalised household income:** |  |  |  |  | *** |  |  |  |  | *** |  |  |  | *** |  |  |  | *** |
| Quintile 1 (lowest) | 15.5 | 5.3 | 4.0 | 6.5 |  | 11.8 | 4.5 | 2.6 | 3.3 |  | 4.9 | 5.5 | 10.5 |  | 6.9 | 6.8 | 11.2 |  |
| Quintile 2 | 20.2 | 13.8 | 10.7 | 8.5 |  | 20.7 | 10.6 | 7.8 | 7.1 |  | 12.3 | 11.8 | 16.2 |  | 14.4 | 13.0 | 19.5 |  |
| Quintile 3 | 21.0 | 22.8 | 18.3 | 16.6 |  | 23.4 | 21.0 | 15.6 | 11.2 |  | 21.1 | 18.0 | 22.6 |  | 21.3 | 19.0 | 22.2 |  |
| Quintile 4 | 20.5 | 29.3 | 27.6 | 24.9 |  | 23.7 | 30.8 | 27.4 | 20.9 |  | 26.8 | 27.3 | 26.0 |  | 27.9 | 25.2 | 23.6 |  |
| Quintile 5 (highest) | 22.8 | 28.8 | 39.4 | 43.5 |  | 20.4 | 33.1 | 46.6 | 57.5 |  | 34.9 | 37.3 | 24.7 |  | 29.5 | 36.0 | 23.5 |  |
| **Chronic illness:** |  |  |  |  | *** |  |  |  |  | *** |  |  |  | * |  |  |  | * |
| None diagnosed | 73.8 | 81.2 | 82.4 | 81.1 |  | 80.8 | 84.5 | 85.5 | 83.1 |  | 79.8 | 79.7 | 83.2 |  | 81.5 | 83.8 | 84.5 |  |
| Diagnosed | 26.2 | 18.8 | 17.6 | 18.9 |  | 19.2 | 15.5 | 14.5 | 16.9 |  | 20.2 | 20.3 | 16.8 |  | 18.5 | 16.2 | 15.5 |  |
| **Smoker status:** |  |  |  |  | *** |  |  |  |  |  |  |  |  | *** |  |  |  | *** |
| Non-smoker | 34.7 | 41.1 | 40.1 | 36.0 |  | 45.4 | 46.7 | 45.0 | 44.8 |  | 41.8 | 38.1 | 37.5 |  | 48.5 | 44.2 | 40.6 |  |
| Ex-smoker | 43.7 | 36.9 | 37.7 | 36.5 |  | 34.8 | 32.3 | 33.0 | 37.6 |  | 38.0 | 39.2 | 36.2 |  | 33.8 | 35.8 | 30.9 |  |
| Smoker | 21.6 | 22.1 | 22.2 | 27.4 |  | 19.9 | 21.0 | 22.1 | 17.6 |  | 20.2 | 22.7 | 26.4 |  | 17.7 | 20.0 | 28.5 |  |
| **Income satisfaction:** |  |  |  |  | ** |  |  |  |  | *** |  |  |  | *** |  |  |  | *** |
| Satisfied | 56.3 | 58.3 | 63.2 | 62.5 |  | 56.3 | 58.8 | 64.1 | 66.5 |  | 61.7 | 61.7 | 55.6 |  | 60.6 | 59.8 | 53.1 |  |
| Neutral | 13.3 | 14.0 | 12.7 | 12.2 |  | 12.6 | 11.2 | 10.7 | 7.3 |  | 12.2 | 13.5 | 14.0 |  | 10.8 | 11.8 | 13.6 |  |
| Dissatisfied | 30.4 | 27.7 | 24.1 | 25.3 |  | 31.1 | 30.0 | 25.2 | 26.2 |  | 26.0 | 24.8 | 30.4 |  | 28.7 | 28.4 | 33.3 |  |
| **Job physicality** |  |  |  |  | *** |  |  |  |  | *** |  |  |  | *** |  |  |  | *** |
| Not at all | 24.8 | 23.0 | 24.3 | 31.6 |  | 21.5 | 16.2 | 18.8 | 27.6 |  | 18.0 | 24.8 | 34.6 |  | 14.2 | 20.7 | 32.4 |  |
| Not very | 41.8 | 35.8 | 37.0 | 35.0 |  | 43.4 | 35.3 | 36.9 | 43.1 |  | 34.0 | 37.2 | 41.1 |  | 36.6 | 40.5 | 47.5 |  |
| Fairly | 20.5 | 24.9 | 25.8 | 23.9 |  | 21.6 | 30.0 | 30.7 | 18.3 |  | 28.5 | 25.2 | 17.1 |  | 29.9 | 25.5 | 14.4 |  |
| Very physical | 12.9 | 16.4 | 12.9 | 9.5 |  | 13.5 | 18.5 | 13.6 | 10.9 |  | 19.5 | 12.8 | 7.2 |  | 19.4 | 13.2 | 5.7 |  |
| **Job satisfaction:** |  |  |  |  |  |  |  |  |  |  |  |  |  | * |  |  |  | ** |
| Satisfied | 78.4 | 75.6 | 78.1 | 80.6 |  | 79.6 | 80.4 | 80.0 | 78.2 | * | 75.3 | 79.4 | 77.3 |  | 80.9 | 80.1 | 77.0 |  |
| Neutral | 7.9 | 8.3 | 7.5 | 6.6 |  | 7.0 | 6.0 | 4.4 | 6.2 |  | 8.7 | 6.8 | 8.1 |  | 5.7 | 5.8 | 8.2 |  |
| Dissatisfied | 13.8 | 16.1 | 14.4 | 12.9 |  | 13.4 | 13.6 | 15.6 | 15.5 |  | 16.0 | 13.9 | 14.6 |  | 13.4 | 14.2 | 14.8 |  |
| **Work autonomy:** |  |  |  |  | *** |  |  |  |  | *** |  |  |  | *** |  |  |  | ** |
| Mean (95%CI) | 11.0 | 11.5 | 11.9 | 12.6 |  | 10.3 | 11.1 | 11.4 | 11.9 |  | 11.6 | 12.1 | 11.2 |  | 10.8 | 11.1 | 10.4 |  |
|  | 10.7 | 11.3 | 11.8 | 12.3 |  | 10.2 | 10.9 | 11.2 | 11.4 |  | 11.5 | 11.9 | 11.0 |  | 10.7 | 10.9 | 10.2 |  |

*Notes: figures are percentages unless stated otherwise. Percentages are weighted. Sample sizes are unweighted. *p<0.05 **p<0.01 ***p<0.001*

**Table S2.** Unadjusted mean depressive symptoms for work arrangements and covariates for men and women (results for complete case analysis)

|  | **Men** | **Women** |
| --- | --- | --- |
| **TEMPORAL WORK PATTERNS** | **Mean GHQ-12 (95%CI)** | **Mean GHQ-12 (95%CI)** |
| **Weekly work hours (hr/wk):** |  |  |
| <35 | 10.2 (9.9, 10.5) | 11.0 (10.9, 11.2) |
| 35-40 ^†^ | 10.1 (9.9, 10.3) | 11.0 (10.8, 11.2) |
| 41-54 | 10.0 (9.8, 10.1) | 11.2 (10.9, 11.5) |
| ≥55 | 10.2 (9.9, 10.5) | 11.8 (11.1, 12.5)* |
| **Weekend work:** |  |  |
| No weekends ^†^ | 9.9 (9.7, 10.1) | 10.9 (10.7, 11.1) |
| Some weekends | 10.2 (10.0, 10.3)* | 11.1 (10.9, 11.3) |
| Most/all weekends | 10.1 (9.9, 10.4) | 11.6 (11.3, 11.9)*** |
| **COVARIATES** |  |  |
| **Age (years):** |  |  |
| 16-34 | 9.8 (9.6, 10.0)** | 10.8 (10.6, 11.0)** |
| ≥35^†^ | 10.2 (10.1, 10.3) | 11.2 (11.1, 11.3) |
| **Marital status:** |  |  |
| Single | 9.8 (9.5, 10.1)* | 11.0 (10.6, 11.3) |
| Married^†^ | 10.1 (10.0, 10.2) | 11.0 (10.9, 11.1) |
| Separated/divorced/widowed | 10.4 (9.9, 10.8) | 11.8 (11.5, 12.2)*** |
| **Children in the household:** |  |  |
| None ^†^ | 10.0 (9.8, 10.1) | 11.0 (10.8, 11.1) |
| Aged 0-4 years | 10.0 (9.7, 10.2) | 11.3 (11.0, 11.6) |
| Aged 5-9 years | 10.4 (10.1, 10.8)* | 11.1 (10.7, 11.4) |
| Aged 10-15 years | 10.6 (10.2, 10.9)*** | 11.4 (11.1, 11.7)* |
| **Educational attainment:** |  |  |
| Degree (or higher) ^†^ | 10.2 (10.1, 10.4) | 11.0 (10.8, 11.2) |
| A levels (or equivalent) | 10.1 (9.9, 10.4) | 10.9 (10.7, 11.2) |
| GCSEs (or equivalent) | 9.9 (9.6, 10.1)* | 11.4 (11.1, 11.6)* |
| Other qualification | 9.9 (9.6, 10.2) | 11.5 (11.1, 11.9)* |
| No qualifications | 9.6 (9.1, 10.1)* | 10.9 (10.4, 11.4) |
| **NS-SEC occupations:** |  |  |
| Managerial/professional ^†^ | 10.1 (10.0, 10.3) | 11.1 (10.9, 11.3) |
| Intermediate | 10.2 (10.0, 10.5) | 10.9 (10.7, 11.2) |
| Routine | 9.9 (9.7, 10.1)* | 11.1 (10.9, 11.4) |
| **Equivalised household income:** |  |  |
| 1^st^ quintile | 10.7 (10.2, 11.2)** | 11.6 (11.2, 12.1)** |
| 2^nd^ quintile | 10.2 (9.9, 10.5) | 11.5 (11.2, 11.8)*** |
| 3^rd^ quintile | 10.1 (9.9, 10.4) | 11.0 (10.7, 11.2) |
| 4^th^ quintile | 10.0 (9.8, 10.2) | 11.0 (10.8, 11.3) |
| 5^th^ quintile (highest amount) ^†^ | 9.9 (9.7, 10.1) | 10.9 (10.7, 11.1) |
| **Chronic illness:** |  |  |
| Not diagnosed^†^ | 10.0 (9.8, 10.1) | 11.0 (10.8, 11.1) |
| Diagnosed | 10.5 (10.3, 10.7)*** | 11.7 (11.4, 12.0)*** |
| **Smoker status:** |  |  |
| Non-smoker ^†^ | 9.9 (9.7, 10.1) | 10.7 (10.6, 10.9) |
| Ex-smoker | 10.0 (9.9, 10.2) | 11.1 (10.9, 11.3)** |
| Smoker | 10.4 (10.2, 10.7)*** | 11.9 (11.6, 12.2)*** |
| **PSYCHOSOCIAL WORK CONDITIONS** |  |  |
| **Satisfaction with income:** |  |  |
| Satisfied ^†^ | 9.2 (9.0, 9.3) | 10.0 (9.9, 10.1) |
| Neutral satisfaction | 10.2 (9.9, 10.5)*** | 11.6 (11.2, 11.9)*** |
| Dissatisfied | 12.1 (11.8, 12.3)*** | 13.1 (12.8, 13.3)*** |
| **Job physicality:** |  |  |
| Not at all physical^†^ | 9.7 (9.5, 10.0) | 11.0 (10.7, 11.3) |
| Not very physical | 10.0 (9.8, 10.1) | 11.0 (10.8, 11.2) |
| Fairly physical | 10.3 (10.1, 10.5)*** | 11.1 (10.8, 11.3) |
| Very physical | 10.5 (10.2, 10.8)*** | 11.4 (11.1, 11.7) |
| **Job satisfaction:** |  |  |
| Satisfied^†^ | 9.5 (9.4, 9.6) | 10.5 (10.3, 10.6) |
| Neutral satisfaction | 11.2 (10.8, 11.6)*** | 12.7 (12.2. 13.2)*** |
| Dissatisfied | 12.7 (12.3, 13.0)*** | 14.0 (13.6,14.3)*** |
| Notes: † denotes reference category. Means are weighted. *P<0.05 **P<0.01 ***P<0.001 | | |

| **Table S3.** Associations between weekly work hours and depressive symptoms (results for complete case analysis) | | | | | | | | | | | | | | |
| --- | --- | --- | --- | --- | --- | --- | --- | --- | --- | --- | --- | --- | --- | --- |
|  | | **Men** | | | | | | | **Women** | | | | | |
|  | | (1) age | | | (2) + SEP + health† | | (3) + mediators‡ | | (1) age | | (2) + SEP + health† | | (3) + mediators‡ | |
|  | | Coef. | | 95% CI | Coef. | 95% CI | Coef. | 95% CI | Coef. | 95% CI | Coef. | 95% CI | Coef. | 95% CI |
| **Work schedule (reference: 35-40 hr/wk)** | | | | | | | | |  |  |  |  |  |  |
| <35 hr/wk | 0.4* | | 0.1, 0.8 | | 0.3 | -0.1, 0.7 | 0.3 | -0.1, 0.6 | 0.1 | -0.2, 0.3 | -0.1 | -0.3, 0.2 | -0.1 | -0.3, 0.2 |
| 41-54 hr/wk | -0.2 | | -0.4, 0.1 | | -0.1 | -0.4, 0.1 | 0.004 | -0.2, 0.2 | 0.2 | -0.2, 0.5 | 0.2 | -0.1, 0.6 | 0.3 | -0.1, 0.6 |
| ≥55 hr/wk | 0.01 | | -0.4, 0.4 | | 0.03 | -0.3, 0.4 | 0.3 | -0.1, 0.6 | 0.7* | 0.02, 1.5 | 0.8* | 0.1, 1.5 | 0.8* | 0.1, 1.5 |
| **Age (continuous)** | | | | | | | | |  |  |  |  |  |  |
| Age | 0.2*** | | 0.2, 0.3 | | 0.2*** | 0.2, 0.3 | 0.1*** | 0.1, 0.2 | 0.2*** | 0.1, 0.2 | 0.2*** | 0.1, 0.2 | 0.1*** | 0.1, 0.2 |
| Age2 | -0.003*** | | -0.003,  -0.002 | | -0.003*** | -0.003,  -0.002 | -0.002*** | -0.002,  -0.001 | -0.002*** | -0.003,  -0.001 | -0.002*** | -0.003,  -0.001 | -0.001*** | -0.002,  -0.001 |
| **Marital status (reference: married)** | | | | | | | | |  |  |  |  |  |  |
| Single/never married | | | | | -0.1 | -0.4, 0.3 | -0.06 | -0.4, 0.3 |  |  | 0.2 | -0.2, 0.6 | 0.03 | -0.3, 0.4 |
| Separated/divorced/widowed | | | | | 0.1 | -0.3, 0.6 | 0.06 | -0.3, 0.5 |  |  | 0.6** | 0.2, 1.0 | 0.2 | -0.2, 0.5 |
| **Children in household (reference: none)** | | | | | | | | |  |  |  |  |  |  |
| 0-4 years | | |  | | -0.3 | -0.7, 0.03 | -0.2 | -0.5, 0.1 |  |  | 0.3 | -0.1, 0.7 | 0.4 | -0.01, 0.7 |
| 5-9 years | | |  | | 0.001 | -0.4, 0.4 | 0.02 | -0.4, 0.4 |  |  | -0.1 | -0.5, 0.3 | 0.03 | -0.4, 0.4 |
| 10-15 years | | |  | | 0.1 | -0.3, 0.5 | 0.2 | -0.2, 0.6 |  |  | 0.07 | -0.3, 0.4 | 0.2 | -0.2, 0.5 |
| **Education attainment (reference: degree)** | | | | | | | | |  |  |  |  |  |  |
| A level | | |  | | -0.1 | -0.4, 0.2 | -0.1 | -0.4, 0.2 |  |  | 0.03 | -0.3, 0.3 | -0.05 | -0.4, 0.3 |
| GCSE | | |  | | -0.5** | -0.8, -0.1 | -0.4** | -0.7, -0.1 |  |  | 0.3 | -0.1, 0.6 | 0.2 | -0.1, 0.5 |
| Other qualification | | |  | | -0.5** | -0.9, -0.1 | -0.5** | -0.9, -0.1 |  |  | 0.4 | -0.1, 0.9 | 0.4 | -0.1, 0.8 |
| No qualifications | | |  | | -0.8** | -1.4, -0.2 | -0.6* | -1.1, -0.1 |  |  | -0.1 | -0.7, 0.5 | -0.05 | -0.6, 0.5 |
| **NS-SEC (reference: manager/professional)** | | | | | | | | |  |  |  |  |  |  |
| Intermediate | | |  | | 0.1 | -0.2, 0.4 | 0.1 | -0.2, 0.4 |  |  | -0.2 | -0.5, 0.1 | -0.3* | -0.6, -0.01 |
| Routine | | |  | | -0.2 | -0.5, 0.09 | -0.5** | -0.8, -0.1 |  |  | -0.2 | -0.5, 0.2 | -0.3* | -0.7, -0.01 |
| **Equivalised household income (reference: 5^th^ quintile)** | | | | | | | | |  |  |  |  |  |  |
| 4^th^ quintile | | |  | | 0.2 | -0.1, 0.5 | -0.2 | -0.5, 0.1 |  |  | 0.2 | -0.2, 0.5 | -0.1 | -0.4, 0.1 |
| 3^rd^ quintile | | |  | | 0.5** | 0.1, 0.8 | -0.1 | -0.4, 0.2 |  |  | 0.01 | -0.3, 0.4 | -0.5** | -0.8, -0.2 |
| 2^nd^ quintile | | |  | | 0.5** | 0.2, 0.9 | -0.2 | -0.6, 0.1 |  |  | 0.6** | 0.1, 1.0 | -0.1 | -0.5, 0.3 |
| 1^st^ quintile (lowest) | | |  | | 0.9*** | 0.4, 1.5 | 0.2 | -0.3, 0.7 |  |  | 0.6* | 0.1, 1.2 | -0.1 | -0.6, 0.5 |
| **Smoker status (reference: non-smoker)** | | | | | | | | |  |  |  |  |  |  |
| Ex-smoker | | |  | | 0.2 | -0.1, 0.4 | 0.1 | -0.1, 0.4 |  |  | 0.3** | 0.1, 0.6 | 0.2 | -0.1, 0.4 |
| Smoker | | |  | | 0.6*** | 0.3, 0.9 | 0.4* | 0.1, 0.7 |  |  | 1.0*** | 0.7, 1.4 | 0.6*** | 0.3, 0.9 |
| **Chronic illness (reference: none diagnosed)** | | | | | | | | |  |  |  |  |  |  |
| Diagnosed condition | | | | | 0.6*** | 0.4, 0.9 | 0.5*** | 0.3, 0.8 |  |  | 0.8*** | 0.5, 1.1 | 0.6*** | 0.3, 0.9 |
| **Income satisfaction (reference: satisfied)** | | | | | | | | |  |  |  |  |  |  |
| Neutral | | | | |  |  | 0.9*** | 0.6, 1.2 |  |  |  |  | 1.4*** | 1.1, 1.8 |
| Dissatisfied | | | | |  |  | 2.4*** | 2.1, 2.7 |  |  |  |  | 2.7*** | 2.4, 3.0 |
| **Job physicality (reference: not at all physical)** | | | | | | | | |  |  |  |  |  |  |
| Not very physical | | | | |  |  | 0.2 | -0.1, 0.4 |  |  |  |  | 0.2 | -0.1, 0.5 |
| Fairly physical | | | | |  |  | 0.5** | 0.1, 0.8 |  |  |  |  | 0.2 | -0.1, 0.5 |
| Very physical | | | | |  |  | 0.4* | 0.01, 0.8 |  |  |  |  | 0.4* | 0.03, 0.8 |
| **Job satisfaction (reference: satisfied)** | | | | | | | | |  |  |  |  |  |  |
| Neutral | | |  | |  |  | 1.2*** | 0.8, 1.6 |  |  |  |  | 1.8*** | 1.3, 2.3 |
| Dissatisfied | | |  | |  |  | 2.5*** | 2.1, 2.8 |  |  |  |  | 2.9*** | 2.5, 3.3 |
| **Work autonomy (continuous)** | | | | | | | | |  |  |  |  |  |  |
| Work autonomy | | |  | |  |  | -0.1*** | -0.1, -0.1 |  |  |  |  | -0.1*** | -0.1, -0.03 |
| Constant | | 5.4*** | | 4.3, 6.6 | 5.4*** | 4.1, 6.7 | 6.6*** | 5.3, 7.9 | 7.5*** | 6.3, 8.7 | 6.7*** | 5.2, 8.2 | 7.3*** | 5.9, 8.8 |
| Survey weights are applied. **p<0.05 **p<0.01 ***p<0.001*  † Model is adjusted for age, age2, marital status, children in household, education attainment, NS-SEC, equivalised household income, smoker status and chronic illness  ‡ Model is also adjusted for income satisfaction, job physicality, job satisfaction, and work autonomy | | | | | | | | | | | | | | |

| **Table S4. Associations between weekend working and depressive symptoms (results for complete case analysis)** | | | | | | | | | | | | | |
| --- | --- | --- | --- | --- | --- | --- | --- | --- | --- | --- | --- | --- | --- |
|  | (1) age | | (2) + SEP + health† | | (3) + mediators‡ | | (1) age | | (2) + SEP + health† | | (3) + mediators‡ | | |
|  | Coef. | 95% CI | Coef. | 95% CI | Coef. | 95% CI | Coef. | 95% CI | Coef. | 95% CI | Coef. | 95% CI | |
| **Work schedule (reference: no weekends)** | | | | | | |  |  |  |  |  |  | |
| Some weekends | 0.2 | -0.03, 0.5 | 0.2 | -0.1, 0.5 | 0.4** | 0.2, 0.6 | 0.2 | -0.1, 0.4 | 0.2 | -0.1, 0.4 | 0.2 | | -0.1, 0.4 |
| Most/all weekends | 0.3 | -0.04, 0.6 | 0.2 | -0.1, 0.5 | 0.4** | 0.1, 0.6 | 0.8*** | 0.4, 1.1 | 0.6*** | 0.3, 1.0 | 0.6*** | | 0.3, 0.9 |
| **Age (continuous)** | | | | | | |  |  |  |  |  | |  |
| Age | 0.2*** | 0.2, 0.3 | 0.2*** | 0.1, 0.3 | 0.1*** | 0.1, 0.2 | 0.2*** | 0.1, 0.2 | 0.2*** | 0.1, 0.3 | 0.1*** | | 0.1, 0.2 |
| age2 | -0.002*** | -0.003, -0.002 | -0.002*** | -0.003, -0.002 | -0.001*** | -0.002,  -0.001 | -0.002*** | -0.003,  -0.001 | -0.002*** | -0.003,  -0.001 | -0.001*** | | -0.002,  -0.001 |
| **Marital status (reference: married)** | | | | | | |  |  |  |  |  |  | |
| Single/never married | |  | -0.1 | -0.4, 0.3 | -0.1 | -0.4, 0.3 |  |  | 0.2 | -0.2, 0.6 | 0.1 | -0.3, 0.4 | |
| Separated/divorced/widowed | | | 0.1 | -0.3, 0.6 | 0.04 | -0.4, 0.5 |  |  | 0.6*** | 0.3, 1.0 | 0.2 | -0.1, 0.5 | |
| **Children in household (reference: none)** | | | | | | |  |  |  |  |  |  | |
| 0-4 years | |  | -0.3 | -0.7, 0.02 | -0.2 | -0.6, 0.1 |  |  | 0.3 | -0.1, 0.6 | 0.3 | -0.1, 0.7 | |
| 5-9 years | |  | 0.001 | -0.4, 0.4 | 0.01 | -0.4, 0.4 |  |  | -0.1 | -0.5, 0.3 | -0.01 | -0.4, 0.4 | |
| 10-15 years | |  | 0.1 | -0.3, 0.5 | 0.2 | -0.2, 0.5 |  |  | 0.05 | -0.3, 0.4 | 0.2 | -0.2, 0.5 | |
| **Education attainment (reference: degree)** | | | | | | |  |  |  |  |  |  | |
| A level | |  | -0.1 | -0.4, 0.2 | -0.1 | -0.4, 0.2 |  |  | 0.01 | -0.3, 0.3 | -0.07 | -0.4, 0.2 | |
| GCSE | |  | -0.5** | -0.8, -0.2 | -0.4** | -0.8, -0.1 |  |  | 0.3 | -0.1, 0.6 | 0.2 | -0.1, 0.5 | |
| Other qualification | |  | -0.6** | -1.0, -0.2 | -0.5** | -0.9, -0.1 |  |  | 0.4 | -0.1, 0.9 | 0.4 | -0.1, 0.8 | |
| No qualifications | |  | -0.8** | -1.4, -0.3 | -0.6* | -1.1, -0.1 |  |  | -0.1 | -0.7, 0.5 | -0.07 | -0.6, 0.5 | |
| **NS-SEC (reference: manager/professional)** | | | | | | |  |  |  |  |  |  | |
| Intermediate | |  | 0.08 | -0.2, 0.4 | 0.1 | -0.2, 0.4 |  |  | -0.2 | -0.6, 0.06 | -0.3* | -0.6, -0.1 | |
| Routine | |  | -0.2 | -0.5, 0.08 | -0.5** | -0.8, -0.1 |  |  | -0.3 | -0.6, 0.04 | -0.4* | -0.8, -0.1 | |
| **Equivalised household income (reference 5^th^ quintile)** | | | | | | |  |  |  |  |  |  | |
| 4^th^ quintile | |  | 0.3 | -0.03, 0.5 | -0.2 | -0.5, 0.1 |  |  | 0.1 | -0.2, 0.4 | -0.2 | -0.5, 0.1 | |
| 3^rd^ quintile | |  | 0.5** | 0.2, 0.8 | -0.1 | -0.4, 0.2 |  |  | -0.1 | -0.4, 0.3 | -0.5*** | -0.9, -0.2 | |
| 2^nd^ quintile | |  | 0.6** | 0.2, 1.0 | -0.2 | -0.6, 0.2 |  |  | 0.5* | 0.1, 0.9 | -0.2 | -0.6, 0.2 | |
| 1^st^ quintile (lowest) | |  | 1.0*** | 0.5, 1.6 | 0.2 | -0.2, 0.7 |  |  | 0.5 | -0.02, 1.1 | -0.2 | -0.7, 0.3 | |
| **Smoker status (reference: non-smoker)** | | | | | | |  |  |  |  |  |  | |
| Ex-smoker | |  | 0.2 | -0.1, 0.4 | 0.1 | -0.1, 0.3 |  |  | 0.3** | 0.1, 0.6 | 0.2 | -0.1, 0.4 | |
| Smoker | |  | 0.6*** | 0.3, 0.9 | 0.4* | 0.1, 0.6 |  |  | 1.0*** | 0.7, 1.3 | 0.6*** | 0.3, 0.9 | |
| **Chronic illness (reference: none diagnosed)** | | | | | | |  |  |  |  |  |  | |
| Diagnosed condition | |  | 0.6*** | 0.4, 0.9 | 0.5*** | 0.3, 0.8 |  |  | 0.8*** | 0.5, 1.1 | 0.6*** | 0.3, 0.9 | |
| **Income satisfaction (reference: satisfied)** | | | | | | |  |  |  |  |  |  | |
| Neutral | |  |  |  | 0.9*** | 0.6, 1.2 |  |  |  |  | 1.4*** | 1.0, 1.8 | |
| Dissatisfied | |  |  |  | 2.4*** | 2.1, 2.7 |  |  |  |  | 2.7*** | 2.4, 3.0 | |
| **Job physicality (reference: not at all physical)** | | | | | | |  |  |  |  |  |  | |
| Not very physical | |  |  |  | 0.2 | -0.1, 0.5 |  |  |  |  | 0.2 | -0.1, 0.5 | |
| Fairly physical | |  |  |  | 0.5** | 0.2, 0.8 |  |  |  |  | 0.3 | -0.1, 0.6 | |
| Very physical | |  |  |  | 0.5* | 0.1, 0.9 |  |  |  |  | 0.5* | 0.1, 0.9 | |
| **Job satisfaction (reference: satisfied)** | | | | | | |  |  |  |  |  |  | |
| Neutral | |  |  |  | 1.2*** | 0.8, 1.6 |  |  |  |  | 1.8*** | 1.3, 2.3 | |
| Dissatisfied | |  |  |  | 2.5*** | 2.1, 2.8 |  |  |  |  | 2.9*** | 2.5, 3.3 | |
| **Work autonomy (continuous)** | | | | | | |  |  |  |  |  |  | |
| Work autonomy | |  |  |  | -0.08*** | -0.1, -0.1 |  |  |  |  | -0.1*** | -0.1, -0.03 | |
| Constant | 5.7*** | 4.6, 6.8 | 5.6*** | 4.3, 6.8 | 6.6*** | 5.4, 7.9 | 7.0*** | 5.8, 8.3 | 6.5*** | 5.0, 8.0 | 7.1*** | 5.6, 8.5 | |
| Survey weights are applied. **p<0.05 **p<0.01 ***p<0.001*  † Model is adjusted for age, age2, marital status, children in household, education attainment, NS-SEC, equivalised household income, smoker status and chronic illness  ‡ Model is also adjusted for income satisfaction, job physicality, job satisfaction, and work autonomy | | | | | | | | | | | | | |
